# Humoral immune response to COVID-19 vaccines in onchocerciasis-infected individuals: a field study from Ghana

**DOI:** 10.3389/fimmu.2025.1633187

**Published:** 2025-10-01

**Authors:** Julia Meyer, Ute Klarmann-Schulz, Jennifer Nadal, Marijo Parcina, Linda Batsa Debrah, Jubin Osei-Mensah, Derrick Adu Mensah, Monica Ahiadorme, Vera Serwaa Opoku, Michael Agyemang Obeng, Eunice Kyaakvile Kuutiero, John Opoku, Alexander Yaw Debrah, Achim Hoerauf, Tomabu Adjobimey

**Affiliations:** ^1^ Institute of Medical Microbiology, Immunology and Parasitology (IMMIP), University Hospital Bonn, Bonn, Germany; ^2^ Bonn-Cologne Site, German Center for Infectious Disease Research (DZIF), Bonn, Germany; ^3^ Institute for Medical Biometry, Informatics, and Epidemiology (IMBIE), University Hospital Bonn, Bonn, Germany; ^4^ Department of Clinical Microbiology, School of Medicine and Dentistry, Kwame Nkrumah University of Science and Technology (KNUST), Kumasi, Ghana; ^5^ Kumasi Centre for Collaborative Research in Tropical Medicine (KCCR), Kwame Nkrumah University of Science and Technology (KNUST), Kumasi, Ghana; ^6^ Department of Pathobiology, School of Veterinary Medicine, Kwame Nkrumah University of Science and Technology (KNUST), Kumasi, Ghana; ^7^ Department of Public Health Education, Akenten Appiah-Menka University of Skills Training and Entrepreneurial Development, Mampong, Ghana; ^8^ Department of Medical Laboratory Technology, Royal Ann College of Health, Kumasi, Ghana; ^9^ Faculty of Allied Health Sciences, Kwame Nkrumah University of Science and Technology (KNUST), Kumasi, Ghana; ^10^ Laboratoire de Biologie intégrative pour l’Innovation thérapeutique (BioInov), Faculté des Sciences et Techniques (FAST), Université d’Abomey Calavi, Abomey Calavi, Benin

**Keywords:** COVID-19 vaccination, SARS-CoV-2 seroprevalence, onchocerciasis, immune response, co-infection, soil-transmitted helminths, antibodies, Ghana

## Abstract

**Introduction:**

COVID-19 vaccines are the most successful medical interventions to reduce disease severity and mortality. Various vaccine platforms with unique properties have been developed. The course of the pandemic was contrary to the initial predictions, relatively mild in sub-Saharan Africa. Besides other contributing factors, helminths may also play a role due to their immunomodulatory properties. This study aimed to investigate antibody responses in onchocerciasis-infected individuals after multiple doses of different COVID-19 vaccines, comparing mRNA and vector-based vaccines, accounting for co-infections, and contrasting the findings with those from healthy endemic controls..

**Methods:**

For this purpose, samples of onchocerciasis-infected individuals (n=110) from a larger and ongoing clinical trial were collected during the COVID-19 pandemic (December 2022) in northwestern Ghana. Participants were grouped according to their COVID-19 vaccine status as unvaccinated, incomplete (1 dose), and complete (2 doses). Helminth-specific and SARS-CoV-2-specific antibodies were quantified using ELISA, while a multiplex immunoassay was used to determine SARS-CoV-2 variant-specific neutralizing antibodies. Non-parametric statistical tests including Kruskal-Wallis and Mann-Whitney-U test were used for group comparison.

**Results:**

The data indicated a high SARS-CoV-2 seroprevalence among both, COVID-19 unvaccinated and vaccinated participants, despite the lack of a confirmed infection. Unvaccinated participants (n=44) showed 86% and 77% seropositivity for spike-specific IgA and IgG, respectively. Incompletely (n=22) and completely (n=36) COVID-19 vaccinated groups showed 63% and 50% nucleocapsid seropositivity, indicating viral exposure. The study identified elevated antibody levels following COVID-19 vaccination despite underlying onchocerciasis infection compared to the unvaccinated control group. However, comparable SARS-CoV-2 IgG levels were detected among mRNA and vector-based vaccinated individuals, as well as a higher breakthrough infection rate (NCP-seropositivity) in mRNA vaccinees. Interestingly, active-infected onchocerciasis individuals (Microfilaria positive) showed a reduced vaccine-induced IgG response after complete COVID-19 vaccination. Moreover, helminths may modify the vaccine- or infection-induced SARS-CoV-2 IgG subclass response, as the lack of IgG1 and the expression of IgG4 was associated with microfilaria and soil-transmitted helminth seropositivity.

**Conclusion:**

The study concluded that COVID-19 vaccines trigger a strong humoral immune response despite underlying onchocerciasis infection. However, parasitic status or microfilaria load may dampen vaccine-induced responses. These findings highlight the complexity of investigating vaccine responses in the presence of an undeterminable infection history, co-infections and polyparasitism in helminth-endemic areas and emphasize the need to further explore parasite-immunomodulatory mechanisms on COVID-19 vaccine efficacy for future vaccine development.

## 1 Introduction

The world has fairly recovered from the COVID-19 pandemic, which displayed the most devastating global health emergency of the 21^st^ century, with far-reaching impact across different regions and consequences for almost every area of life (1–8). By now, the 25^th^ March 2025, over 777 million COVID-19 cases and more than 7 million deaths globally were reported to the WHO (9). The clinical presentation of COVID-19 shows a broad range, varying from asymptomatic infections to respiratory failure (10). The majority of infected individuals have mild to moderate symptoms, including fever, cough, shortness of breath, muscle ache, sore throat, fatigue, headache and pneumonia (11–13). However, disease progression and severe cases may result in acute respiratory distress syndrome (ARDS), multiple organ failure, and even death (11–13). Pathological mechanisms in severe cases include a dysregulated immune response followed by ineffective host defense, leading to continuous tissue inflammation and damage (14). A dysregulated immune response often results in a “cytokine storm” which is characterized by the release of high levels of pro-inflammatory cytokines, such as interleukin-6 (IL-6), tumor necrosis factor alpha (TNFα), and interferon-gamma (IFNγ) (15). Several risk factors associated with COVID-19 disease severity have been determined, including advanced age, male sex, obesity, and comorbidities such as diabetes, kidney injury, COPD, and hypertension (16). The case fatality rate of COVID-19 depends on several factors and ranges from 4 to 11% across different countries (17). SARS-CoV-2 enters host cells via the angiotensin-converting enzyme 2 (ACE2) receptor and induces an innate immune response, which results in the production of proinflammatory cytokines (18). During the later phase of an infection, the adaptive immune response is activated, leading not only to B cell maturation and the production of neutralizing antibodies, but also to T cell activation, generation of memory lymphocytes, and the establishment of long-term immunological memory (19). Antibodies or immunoglobulins (Ig) are a key player of the adaptive immune response due to a variety of effector functions, such as neutralization of invading pathogens (20). Immunoglobulin G (IgG) is the most abundant isotype in human serum and can be further divided into four subclasses: IgG1, IgG2, IgG3 and IgG4 (20). Each subclass has unique features and varying abilities to induce effector functions, and therefore differs in pro- or anti-inflammatory potential (20). One of the most powerful medical interventions to reduce COVID-19 severity and mortality is the development of vaccines. Various vaccines based on different strategies have been developed, such as mRNA, vector-based, inactivated virus, and recombinant protein vaccines. The vaccines differ within their properties regarding reactogenicity and immunogenicity. The mRNA vaccines Comirnaty (or BNT162b2) and Spikevax (mRNA-1273), developed by BioNTech/Pfizer and Moderna, encode the full-length spike protein, and are known for their rapid development, possible large-scale production, and strong induction of both T cell and antibody responses (21). The vector-based vaccines Vaxzevria (or AZD1222) and Jcovden (Ad26.COV2.S) which were developed by AstraZeneca and Johnson & Johnson, use a viral adenovirus vector as carrier to transfer the genetic information of the SARS-CoV-2 spike protein (21). On the 26^th^ March 2025,according to Our World in Data, 13.72 billion COVID-19 vaccine doses were administered so far worldwide, with 1.43 billion doses in Europe and 874 million in Africa (22). While 70.7% of the world’s population received at least one vaccine dose, the vaccine coverage in Africa was remarkably lower, with 38.69% of individuals receiving at least one dose (22). Despite low vaccine coverage among African countries, the COVID-19 pandemic has been relatively mild, with a low mortality rate (9). This dissimilarity raises questions and emphasizes the need for further research on contributing factors. According to speculations, a younger population, diverse genetic backgrounds, and the absence of diagnostic tests could have contributed to the unpredicted mild pandemic in Africa (23). Aside from these, helminth infections might play a crucial role in mitigating COVID-19 severity (24). A negative association between *Ascaris* seropositivity and COVID-19 severity has already been shown (25). Moreover, it was shown that helminth seropositivity inversely correlated with Th1 and Th17 cytokines and severe COVID-19 in Ghanaian individuals (26). In addition, a reduced activation of SARS-CoV-2-specific CD4^+^ T cells has been shown *in-vitro*, in the presence of worm antigens (27). The immunomodulatory effects of helminths might conflict those with of the host in terms of disease severity and vaccine efficacy. Helminths may potentially lead to reduced inflammation, often related to disease severity, thereby decreasing pathology and associated mortality (28, 29). However, on the other side, helminths infection might lead to a reduced vaccine efficacy (30). Impaired cellular and humoral responses towards other vaccines like Tetanus, Bacillus Calmette Guerin (BCG), or Rubella have already been shown for filarial infections such as Lymphatic Filariasis and Onchocerciasis (31–36). Nevertheless, a robust antibody response following COVID-19 vaccination has been shown in Lymphatic Filariasis individuals (37). This further highlights the need for ongoing research regarding in-depth investigation of the underlying immunological mechanisms for future vaccine development and the reduction of vaccine hesitancy. Onchocerciasis, also known as river blindness, is an eye and skin disease caused by the filarial nematode *Onchocerca volvulus* (38). The disease is transmitted by blackflies, and its progression can lead to visual impairment and blindness (39). The disease primarily affects rural populations in sub-Saharan Africa, and according to estimates, there are over 14 million people with skin disease and more than 1.1 million people with vision loss (40). Helminths are known for their immunoregulatory mechanisms that ensure the tolerance of the human host and enable their survival (41). This mechanism involves the induction of a helper T cell type 2 (Th2) response and regulatory T cells (Treg), which are characterized by regulatory cytokines such as transforming growth factor beta (TGF-β) and interleukin -10 (IL-10), which suppress a Th1-driven response (41). In addition to filaria, soil-transmitted helminths (STH) are highly prevalent in tropical regions, mainly affecting rural areas with poor hygiene and sanitation standards (42). STH infections, including *Ascaris lumbricoides* and *Strongyloides stercoralis*, are among the most common infections worldwide, with an estimated number of 1.5 billion infected individuals, accounting for 24% of the world’s population (42). STHs primarily affect the gastrointestinal tract and are often associated with asymptomatic infections. However, a high parasitic load, particularly in children, can result in abdominal discomfort, diarrhea, and malnutrition (42). The interaction between COVID-19 and chronic helminth infections remains poorly understood, and further studies are required to decipher the combined effects on clinical disease outcomes and vaccine efficacy. This highlights the importance of the present study, which investigated the relationship between COVID-19 and onchocerciasis in a helminth-endemic region of Ghana. This study focused on characterizing humoral responses in individuals with onchocerciasis following multiple doses of different COVID-19 vaccines, comparing mRNA- and vector-based platforms, and benchmarking these responses against healthy endemic controls. Moreover, the complex interplay between chronic filarial infections and additional co-infections was evaluated in both COVID-19 vaccinees and those following a natural infection. This study reports important findings and helps unravel possible synergies or conflicts, especially for helminth-endemic regions, where both COVID-19 and onchocerciasis coexist. Analyzing the influence of onchocerciasis on the immune response to distinct COVID-19 vaccines provides helpful information for future vaccine research and public health strategies.

## 2 Material and methods

### 2.1 Study design and clinical characteristics

**Figure 1:**
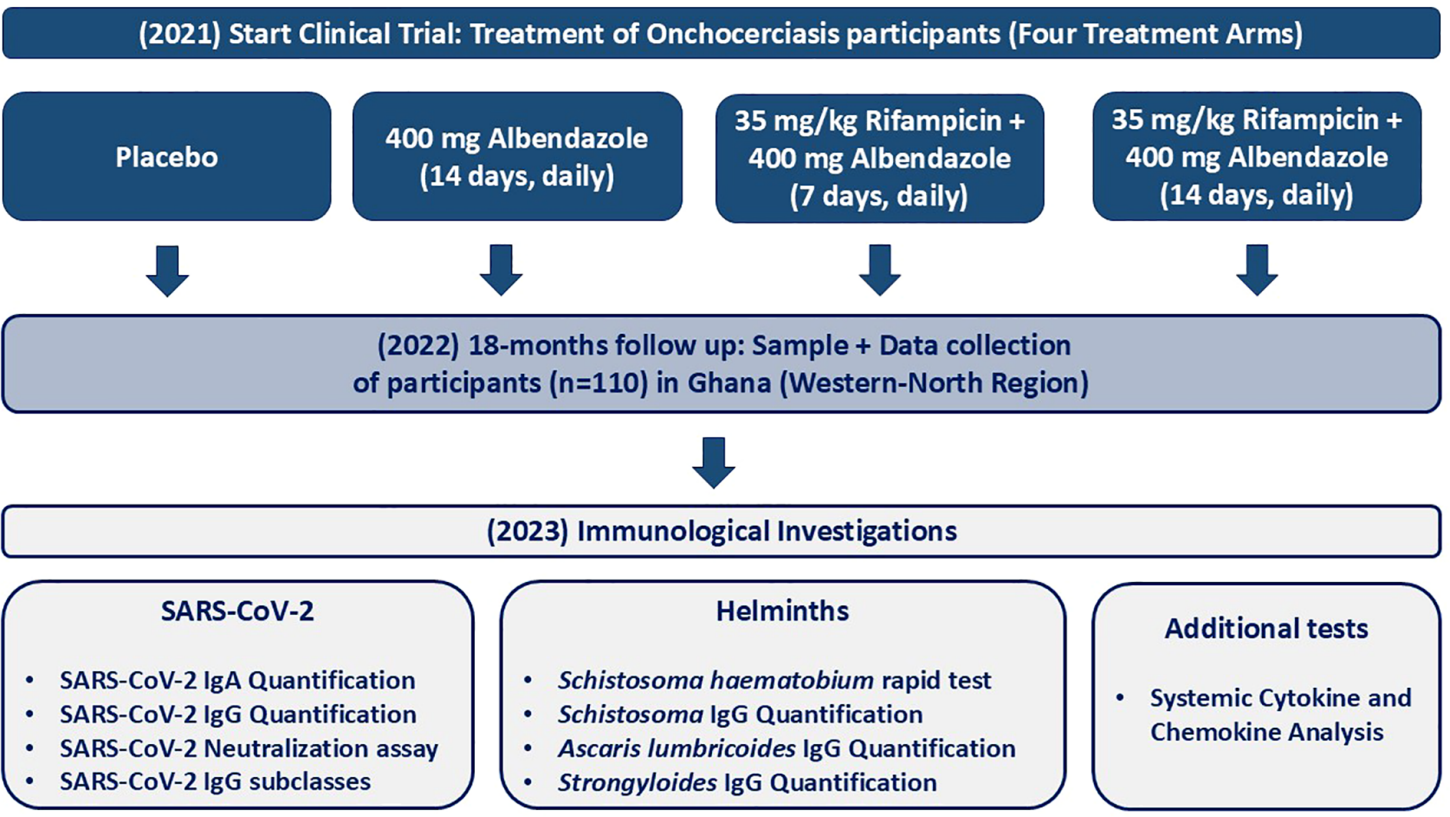
Flowchart of the study design and timeline for assessing the immunological responses in treated onchocerciasis-infected patients. The treatment involved four treatment arms including: (1) Placebo (n=27), (2) 400 mg albendazole (14 days, daily) (n=30), (3) 35 mg/kg rifampicin + 400 mg albendazole (7 days, daily) (n=27) and (2) 35 mg/kg body weight rifampicin + 400 mg albendazole (14 days, daily) (n=26). Blood samples were collected in December 2022 during the COVID-19 pandemic from individuals with onchocerciasis who were part of a treatment trial (started in 2021) in the Western-North region of Ghana. The study included 110 individuals. Demographic and clinical information was collected, and immunological investigations were performed, including SARS-CoV-2 IgA/IgG quantification, SARS-CoV-2 neutralization assay, SARS-CoV-2 IgG subclass quantification, *Schistosoma haematobium* rapid test, *Schistosoma* IgG quantification, *Ascaris* IgG quantification, *Strongyloides* IgG quantification, and systemic cytokine and chemokine analysis. Sample and data collection occurred in Ghana, while immunological investigations (except for the *Schistosoma* rapid test) were conducted at the Institute for Medical Microbiology, Immunology, and Parasitology (IMMIP) in Bonn, Germany.

Blood samples from onchocerciasis-infected participants (n=110) were collected in the northwestern region of Ghana in December 2022. The present study was part of a larger and ongoing clinical trial (“Alternative treatment strategies using anti-wolbachial drugs to accelerate elimination of onchocerciasis and lymphatic filariasis” (ASTAWOL). Ethical approval was obtained from the local ethics committee (approval code: CHRPE/AP/492/20) and ethical board of the University Hospital Bonn (approval code: 439/20). The controlled trial was registered with the following number: TMA2018SF-2451. Each participant provided an informed consent for participation. Samples were collected in Ghana and shipped to the Institute of Medical Microbiology, Immunology, and Parasitology (IMMIP) of the University Hospital of Bonn, where the experiments were performed (**Figure 1**). A total of 110 participants (76 males and 34 females) were included in this study. Participants received treatment between March and April 2021. The participants were divided into four different treatment arms: (1) 35 mg/kg body weight rifampicin + 400 mg albendazole (7 days, daily), (2) 35 mg/kg body weight rifampicin + 400 mg albendazole (14 days, daily), (3) 400 mg albendazole (14 days, daily), and (4) placebo. Samples were taken 18 months after treatment. All participants were excluded for MDA treatment since the beginning of the trial. Palpation for the nodules and parasitological examination of skin biopsies (snips) for microfilaria detection were performed at baseline, 12 months, and 18 months after treatment onset. Skin snips from the right and left iliac were taken from each participant and placed in saline (overnight incubation) for larval migration into the solution. Microfilariae were then counted microscopically. An additional rapid test (ICT Diagnostics, Cape Town, South Africa, #SCH25) for detection of *Schistosoma haematobium* antigen in urine was carried out for the majority of participants (n=101). Active gastrointestinal helminth co-infections were determined via multiplex-PCR in participant’s stool samples. In addition, the following serological tests were used to determine the parasitic status: *Schistosoma* IgG, *Ascaris lumbricoides* IgG, *Strongyloides stercoralis* IgG (**Table 1**). Due to limited COVID-19 test availability and capacity, none of the participants (n=110) had a confirmed SARS-CoV-2 infection (positive test via PCR or rapid antigen test), however COVID-19 related symptoms during the pandemic were well documented using a questionnaire. The following symptoms were included in the questionnaire: loss of appetite, loss of smell, fever, headache, cough, common cold, sore throat, shortness of breath, respiratory problems, fatigue, sweating or chills, musculoskeletal pain, abdominal pain, nausea/vomiting, and chest tightness. The majority (89%) of participants did not exhibit any COVID-19 related symptoms, and none of the participants were hospitalized during the pandemic because of severe COVID-19 or any other clinical condition. All participants received COVID-19 vaccine doses between January 2021 and December 2022. The participants were grouped according to the number of received doses into incompletely (1 dose) and completely (2 doses), regardless of the vaccine type. Vaccine information was well recorded and obtained from participants’ vaccine cards (**Table 1** and **Supplementary Material**). Blood samples of 12 healthy endemic individuals served as control group. The majority (n=10) of healthy endemic controls (HEC) received a COVID-19 vaccination (between March 2021 and August 2021), detailed demographic data is provided in the supplements. HEC samples were collected in December 2021. All HECs were onchocerciasis-negative, without the presence of nodules or viable microfilaria.

**Table 1:** Demographic and clinical characteristics of onchocerciasis participants.

Sample Size (n=)	110			
Age (Min-Max, Mean ± SD)	18 - 55 (38.41 ± 10.4)			
Sex (M / F)	76 (69%) / 34 (31%)			
Weight (kg) (Min-Max, Mean ± SD)	35 – 85 (57.5 ± 8.9)			
Mf in Skin Snips (–/+)	29 (26%) / 81 (74%)			
No. of Mf (Min-Max, Mean ± SD)	1-398 (49.9 ± 76.2)			
No. of Nodules (Min-Max, Mean ± SD)	0 – 14 ( 2.5 ± 1.8)			
COVID-19 positive test (No / Yes)	110 (100%) / 0 (0%)			
COVID-19 vaccination (No / Yes / Unkown)	44 (40%) / 64 (58%) / 2 (2%)			
Dose / COVID-19 vaccine	AstraZeneca (n/%)	J&J (n/%)	mRNA (n/%)	NA (n/%)
Incomplete vaccination: 1. Dose (n=22)	15 / 68%	3 / 14%	2 / 9%	2 / 9%
Complete vaccination: 2. Dose (n=36)	26 / 72%	2 / 6%	8 / 22%	0 / 0%
Booster vaccination 3. Dose (n=6)	1 / 17%	0 / 0%	5 / 83%	0 / 0%
COVID-19 related symptoms (No / Yes)	98 (89%) / 12 (11%)			
Schistosoma haematobium rapid test (n=101) –/+	69 (68%) / 32 ( 32%)			
Schistosoma IgG (n=110) –/+	74 (67%) / 36 (33%)			
Ascaris lumbricoides IgG (n=110) –/+	6 (5%) / 104 (95%)			
Strongyloides stercoralis IgG (n=110) –/+	61 (55%) / 49 (45%)			

SD, Standard Deviation; Mf, Microfilariae; No, Number; NA, not available.

### 2.2 Quantification of SARS-CoV-2 specific antibodies

Enzyme-linked immunosorbent assay (ELISA) Kits (Euroimmun, Lübeck, Germany) were used to detect SARS-CoV-2-Spike-specific IgA/IgG antibodies (#EI2606-9601A/EI2606-9601G) in the plasma of Ghanaian onchocerciasis-infected participants. SARS-CoV-2 Nucleocapsid (NCP)-specific IgG antibody ELISA Kit (Euroimmun, Lübeck Germany, #EI2606-9601-2G) was used to detect NCP-specific antibodies in the plasma of COVID-19 vaccinated participants, according to the manufacturer’s protocol. The samples were automatically diluted 1:101 in the provided sample buffer and incubated for 60 min at 37°C in a pre-coated 96-well plate. Washing and incubation steps were performed automatically using the predesigned program of Euroimmun’s analyzer I automate. Optical density (OD) was measured at 450 nm. Ratios <0.8 were considered negative, 0.8-1.1 as equivocal, and >1.1 as positive.

### 2.3 SARS-CoV-2 IgG-subclass ELISA

SARS-CoV-2 Spike Protein (Thermo Fisher, Waltham, USA, #RP-87700) was diluted (2µg/ml) in coating buffer (Thermo Fisher, Waltham, USA, #00-0044-59), and a high-binding 96- well plate (Greiner, Kremsmünster, Austria, #655061) was coated (50µl/well) and incubated overnight at 4°C. An arbitrary standard of pooled plasma samples of three randomly selected COVID-19 vaccinees was used. The plate was washed 3x with washing buffer and blocked with 200µl/well ELISA buffer (Thermo Fisher, Waltham, USA, #00-4202-56) for 1h at RT. After another washing step, 50 µl/well of diluted sample (1:500 for IgG1, 1:200 for IgG3, IgG4) were added and incubated overnight at 4 °C. The plate was washed and 50µl/well of HRP-conjugated secondary antibodies: anti-human IgG1 (Thermo Fisher, Waltham, USA, #A-10648), anti-human IgG3 (Thermo Fisher, Waltham, USA, #05-3620), anti-human IgG4 (Thermo Fisher, Waltham, USA, #MH1742) were added and incubated for 2h at RT. The plate was washed and 50µl/well of Tetramethylbenzidine (TMB) substrate (Thermo Fisher, Waltham, USA, #00-4201-56) was added and incubated for 15 min. at RT. The reaction was stopped by adding 25µl/well of stop solution (1M H_2_SO_4_). Optical density was measured at 450 nm (Spectramax 190, Molecular Devices, München, Germany). The arbitrary standard allows the semiquantitative detection of SARS-CoV-2 specific IgG subclasses. A cut-off value was not determined; therefore, qualitative analysis was not possible.

### 2.4 Detection of *Ascaris lumbricoides* specific IgG

*Ascaris lumbricoides* IgG ELISA Kit (DRG Instruments GmbH, Marburg, Germany, #EIA-3817) was used to detect specific IgG immunoglobulins in the plasma of onchocerciasis-infected participants and carried out manually according to the manufacturer’s protocol. Briefly, the samples were diluted (1:101) with sample dilution buffer. Samples/standards and controls (100µl) were added to each well of the precoated 96-well plate and incubated for 1h at 37°C. The wells were washed 3x with 300 µl washing solution. 100 µl conjugate was added and incubated for 30 min at RT. After another wash step, 100 µl substrate (TMB) was added and incubated for 15 min at RT in the dark. 100 µl of stop solution was added, and optical density (OD) was measured at 450/620nm. The absorbance was converted into units according to the manufacturer’s protocol with the following formula: (sample absorbance value x 10)/(cut-off) = DRG Units (DU). The results were interpreted as recommended by the manufacturer: >11 DU as positive, 9–11 DU as equivocal, and <9 DU as negative. The diagnostic specificity is 95%, and the sensitivity is 100%. A cross-reaction with antibodies against *Toxocara canis*, *Trichinella, Fasciola*, *Filaria*, and *Strongyloides* cannot be excluded.

### 2.5 Detection of *Strongyloides*-specific antibodies

*Stongyloides ratti* ELISA-Kit (Bordier Affinity Products, Crissier, Switzerland, #9450) was used for the quantitative detection of IgG immunoglobulins specific for *Strongyloides*-nematode infection, according to the manufacturer’s protocol. Briefly, the pre-coated 96-well plate was blocked with 300 µl buffer for 15 min. at RT. The samples were diluted (1:201) with dilution buffer (1X). 100 µl of samples/controls were added to each well and incubated for 30 min at 37°C. The wells were washed 4x with 300 µl washing solution. 100 µl conjugate was added and incubated for 30 min at 37nbsp;°C. After another wash step, 100 µl substrate was added and incubated for 30 min at 37°C. 100 µl of stop solution was added, and optical density (OD) was measured at 450nm. The absorbance was converted into units according to the manufacturer’s protocol with the following formula: (sample absorbance value)/(cut-off absorbance value) = Index. Results were interpreted as recommended by the manufacturer: >1.0 Index as positive and <1.0 as negative. The diagnostic specificity is given with 96%, and the sensitivity with 90%. Cross-reactivity occurred with bilharzia, filariasis, toxocariasis, fasciolosis, and amebiasis.

### 2.6 *Schistosoma*-IgG ELISA

*Schistosoma mansoni* IgG ELISA (Euroimmun, Lübeck, Germany, #EI2300-9601G) was used for the semiquantitative detection of human IgG immunoglobulins specific for *Schistosoma* spp. in plasma or serum. The assays were carried out manually according to the manufacturer’s protocol. Samples were diluted 1:101 in the provided sample buffer and incubated for 60 min at 37°C in a pre-coated 96-well plate. Each washing step was performed three times with 300 µl washing buffer (1X). 100 µl/well conjugate (peroxidase-linked anti-human IgG) was incubated for 30 min at 37°C. Lastly, 100 µl/well substrate solution was added and incubated for 30 min at RT. The reaction was stopped with 100 µl/well stop solution, optical densities (OD) were measured at 450 nm. Ratios <0.8 were considered negative, 0.8-1.1 as equivocal, and >1.1 as positive. Cross reactivity with other helminths and protozoa, especially *plasmodium* spp. and filaria cannot be excluded. The diagnostic specificity is given with 79.4-97.8%, while the sensitivity is given with 72.7-93.2%.

### 2.7 Quantification of SARS-CoV-2 neutralizing antibody levels

Neutralizing SARS-CoV-2 variant-specific antibodies were quantified using the SARS-CoV-2 Variants Neutralizing Antibody 6-plex ProcartaPlex Panel (Thermo Fisher, Waltham, USA, #EPX060-16018-901). The assay was performed according to the manufacturer’s instructions and allowed the comparison of neutralizing antibody capacity towards six SARS-CoV-2 variants, including Wildtype (WT), Alpha (B.1.1.7), Beta (B.1.351), Gamma (P.1), Delta (B.1.617.2) and Omicron (B.1.1.529). The principle is that magnetic beads are conjugated with WT or variant proteins, the neutralizing antibodies in the samples will bind to the beads and compete with biotinylated ACE2. Streptavidin-phycoerythrin (PE) is used as detection reagent and the signal is indirectly proportional to the amount of variant-specific neutralizing antibodies. 50 µl of magnetic capture beads were added to each well of the provided 96-well plate and washed with 150µl 1X washing solution. 25 µl of prediluted samples (1:100) were added, followed by 25 µl assay diluent. Positive and negative control was prepared according to the manufacturer’s recommendation and added to the dedicated wells. Samples were incubated for 2h at RT while shaking (500 rpm/minute). After two washing steps, 25 µl of prediluted 1X detection antibody was added to each well and incubated for 30 min at RT while shaking. After another two washing steps, 50 µl of streptavidin-PE solution was added to each well and incubated for another 30 min at RT on a plate shaker. After two more washing steps, 120 µl of reading buffer was added to each well, and the plate was incubated for 5 min while shaking before the plate was measured using the MagPix Luminex instrument. The results were analyzed using the following neutralization equation: [1 – (MFI of samples/MFI of negative control)] x 100 = Neutralization (%).

### 2.8 Quantification of systemic cytokine and chemokine levels

Cytokine storm 21-plex human ProcartaPlex Panel (Thermo Fisher, Waltham, USA, #EPX210-15850-901) was used to quantify systemic cytokines and chemokines in the plasma of Ghanaian onchocerciasis-infected participants. The assay was performed according to the manufacturer’s protocol and in-volved 21 cytokines and markers related to cytokine release syndrome (CRS), including: G-CSF (CSF-3), GM-CSF, IFN alpha, IFNγ, IL-1 beta, IL-2, IL-4, IL-5, IL-6, IL-8 (CXCL8), IL-10, IL-12p70, IL-13, IL-17A (CTLA-8), IL-18, IP-10 (CXCL10), MCP-1 (CCL2), MIP-1 alpha (CCL3), MIP-1 beta (CCL4), TNF alpha, and TNF beta. 50 µl of magnetic capture beads were added to each well of the provided 96-well plate and washed with 150µl 1X washing solution. 25 µl of undiluted samples were added to the dedicated wells, followed by 25 µl assay diluent. Standard and controls were prepared according to the manufacturer’s recommendation, and samples were incubated for 2h at RT while shaking (500 rpm/minute). After two washes, 25 µl of 1X detection antibody was added to each well and incubated for 30 min. at RT while shaking. After two more washing steps, 50 µl of streptavidin-PE solution was added to each well and incubated for another 30 min. at RT on a plate shaker. After the final two washing steps, 120 µl of reading buffer was added to each well and the plate was incubated for 5 min. The plate was then analyzed using the MagPix Luminex instrument, and the results were analyzed using the ProcartaPlex Analyst application (Thermo Fisher).

### 2.9 Allplex™ GI-Helminth (I) polymerase chain reaction (qRCR)

A multiplex real-time-qPCR was used to detect *Enterocytozoon* spp./*Encephalitozoon* spp., *Strongyloides* spp., *Hymenolepsis* spp., *Ascaris* spp., *Taenia* spp., *Trichuris trichiura*, *Ancylostoma* spp., *Enterobius vermicularis*, and *Necator americanus* co-infections in stool samples of Ghanaian onchocerciasis-infected patients. The assay was performed according to the manufacturer’s instructions and allowed a simultaneous amplification and detection of single or multiple gastrointestinal helminth infections. The DNA extraction and preparation for the qPCR were carried out automatically (Microlab Nimbus, Seegene, Seoul, South Korea). Stool samples were transferred in appropriate transport medium using a swab (eSwab™, Copan, Brescia, Italy, #490CE). The tubes were centrifuged and the supernatant was used for the DNA extraction. Internal controls were used for the validation of the extraction, and the identification of potential PCR-inhibitors. A mastermix containing primer, polymerase, deoxyribonucleotide triphosphate (dNTP), and RNase-free water, was prepared and added to the samples automatically. The amplification was performed using the CFX96 Real-Time PCR Detection System (Bio-Rad, California, USA). The cycle threshold (Ct) value was ≤ 45, and Ct values below 45 were considered as positive. Cross reactivity towards 198 other organisms was tested and not identified. The sensitivity showed a detection limit of 100 copies/reaction.

### 2.10 Statistics

Data were analyzed using GraphPad Prism version 10 (La Jolla, CA, USA). We described the characteristics for the study participants using mean ± standard deviation (SD), minimum (min) and maximum (max) for continuous variables. Categorical variables are presented as frequency distributions with percentages. Confounding factors including age, gender and weight were considered and did not show any influence on the COVID-19 vaccine response. The nonparametric Kruskal-Wallis test, followed by Dunn’s *post-hoc* test, was used for group comparisons, along with the Mann-Whitney U-test. Correlations were performed using the nonparametric Spearman rank-order analysis. A p-value <0.05 was considered statistically significant.

## 3 Results

### 3.1 Increased SARS-CoV-2 specific antibody response following COVID-19 vaccination in Ghanaian onchocerciasis-infected individuals

**Figure 2:**
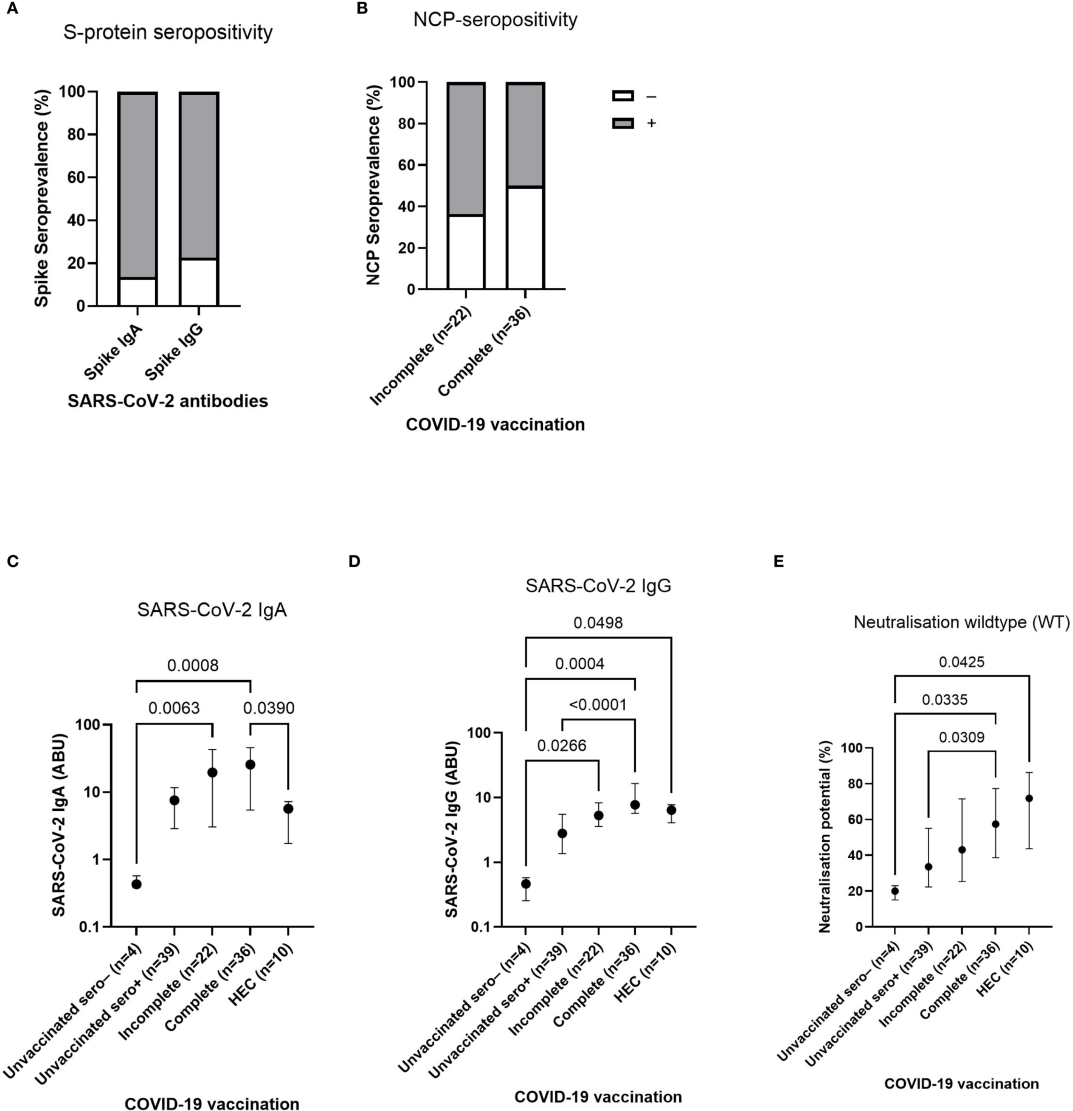
SARS-CoV-2 seroprevalence, antibody response and neutralizing potential in Ghanaian onchocerciasis-infected individuals. **(A, B)** show seropositivity rates for SARS-CoV-2-specific antibodies among COVID-19 unvaccinated **(A)** and vaccinated **(B)** individuals. Bars show 86.36% SARS-CoV-2 spike-specific IgA (n=38) and 77.27% spike-specific IgG (n=34) **(A)** seropositivity (grey) among COVID-19 unvaccinated (n=44) individuals. Within incompletely (1 dose) vaccinated, 63.64% (n=14) were tested positive for nucleocapsid (NCP)-specific IgG (grey), while 50% (n=18) of the fully (2 doses) vaccinated group show NCP-seropositivity **(B)**. White parts represent seronegative groups. **(C, D)** show SARS-CoV-2 spike-specific IgA **(C)** and IgG **(D)** antibody response after COVID-19 vaccination in participants who were grouped according to their COVID-19 vaccination status into: unvaccinated and seronegative (n=4), unvaccinated and seropositive (n=39), incompletely (1 dose) vaccinated (n=22), and fully (2 doses) vaccinated (n=36), compared to vaccinated healthy endemic controls (HEC) (n=10). D shows neutralizing potential of SARS-CoV-2 specific antibodies towards the wildtype **(E)** after COVID-19 vaccination and/or natural infection in comparison to vaccinated HECs. Indicated p-values were calculated using Kruskal-Wallis’ test followed by Dunn’s *post hoc* test for group comparison **(C-E)**. Bars **(A, B)** represent seroprevalence (%) of SARS-CoV-2 antibodies. Dots **(C-E)** represent the median ± IQR of antibody binding units **(C, D)** and neutralizing potential **(E)**. Significance is accepted if p <0.05.

As a result of inadequate COVID-19 test facilities for the Ghanaian population living in the rural areas of the Western-North Region, none of the study participants (n=110) has ever tested positive for SARS-CoV-2. In that particular case, serological tests were used to further examine viral exposure. The analysis revealed, that most COVID-19 unvaccinated participants (n=44) were tested positive for both, SARS-CoV-2 spike-specific IgA (86.36%) and IgG (77.27%) (**Figure 2A**). Since all COVID-19 vaccinated participants received spike-protein based vaccines, the viral nucleocapsid (NCP) protein helps for detection of prior or breakthrough infections. Among incomplete vaccinated (1 dose) individuals (n=22), the majority was tested positive for NCP-IgG (63.64%), while 50% of complete (2 doses) vaccinated (n=36) showed NCP seropositivity (**Figure 2B**). After determination of the seroprevalence among Ghanaian onchocerciasis-infected individuals, the antibody response was quantified to further investigate a potential influence of an underlying onchocerciasis infection onto COVID-19 vaccine efficacy. The Ghanaian onchocerciasis-infected participants were grouped according to their COVID-19 vaccination status into: unvaccinated and seronegative, unvaccinated and seropositive, incompletely vaccinated (1 dose) and completely vaccinated (2 doses). The COVID-19 unvaccinated but SARS-CoV-2 seropositive group, was considered as COVID-19 infected group despite the lack of a confirmed infection by PCR or rapid test. The vaccine response of Ghanaian onchocerciasis-infected participants was compared to COVID-19 vaccinated healthy endemic controls (HEC). A robust SARS-CoV-2-specific antibody response after COVID-19 vaccination has been shown despite an underlying onchocerciasis infection. Incompletely (n=22) and fully (n=36) COVID-19 vaccinated individuals showed elevated SARS-CoV-2 specific IgA and IgG levels compared to the COVID-19 unvaccinated and seronegative control group (**Figures 2C, D**). COVID-19 unvaccinated but SARS-CoV-2 seropositive individuals showed significantly reduced (p<0.0001) IgG levels compared to the fully vaccinated group (**Figure 2B**). Interestingly, significantly reduced SARS-CoV-2 IgA levels were observed in healthy controls (n=10) compared to the completely vaccinated group (**Figure 2C**) and statistical comparable SARS-CoV-2 IgG levels were observed among both groups (**Figure 2D**). Since a robust antibody response cannot necessarily be translated into functionality of antibodies, a neutralization assay was performed to further investigate the quality of antibodies following COVID-19 vaccination and/or natural infection. A cell- and virus-free competitive assay was used to determine the neutralizing potential towards the viral wildtype (WT) and five variants of concern including: Alpha (B.1.1.7), Beta (B.1.351), Gamma (P.1), Delta (B.1.617.2) and Omicron (B.1.1.529). Interestingly, the completely vaccinated individuals and healthy controls showed significantly increased neutralization potential towards the viral wildtype compared to the unvaccinated and seronegative control group (**Figure 2E**). In addition, completely vaccinated individuals showed higher neutralization potential than the COVID-19 infected group (unvaccinated and seropositive). However, no statistical difference within the neutralizing capacity of SARS-CoV-2 antibodies was observed among completely vaccinated onchocerciasis-infected individuals and healthy endemic controls. Statistically comparable neutralizing antibody levels were observed towards all variants among all onchocerciasis-infected participant groups (unvaccinated, incompletely, completely vaccinated) in comparison to the healthy controls (**Supplementary Figure 1**).

### 3.2 Comparison of SARS-CoV-2 antibody response following complete vaccination with mRNA and vector-based COVID-19 vaccines in Ghanaian onchocerciasis-infected individuals

**Figure 3:**
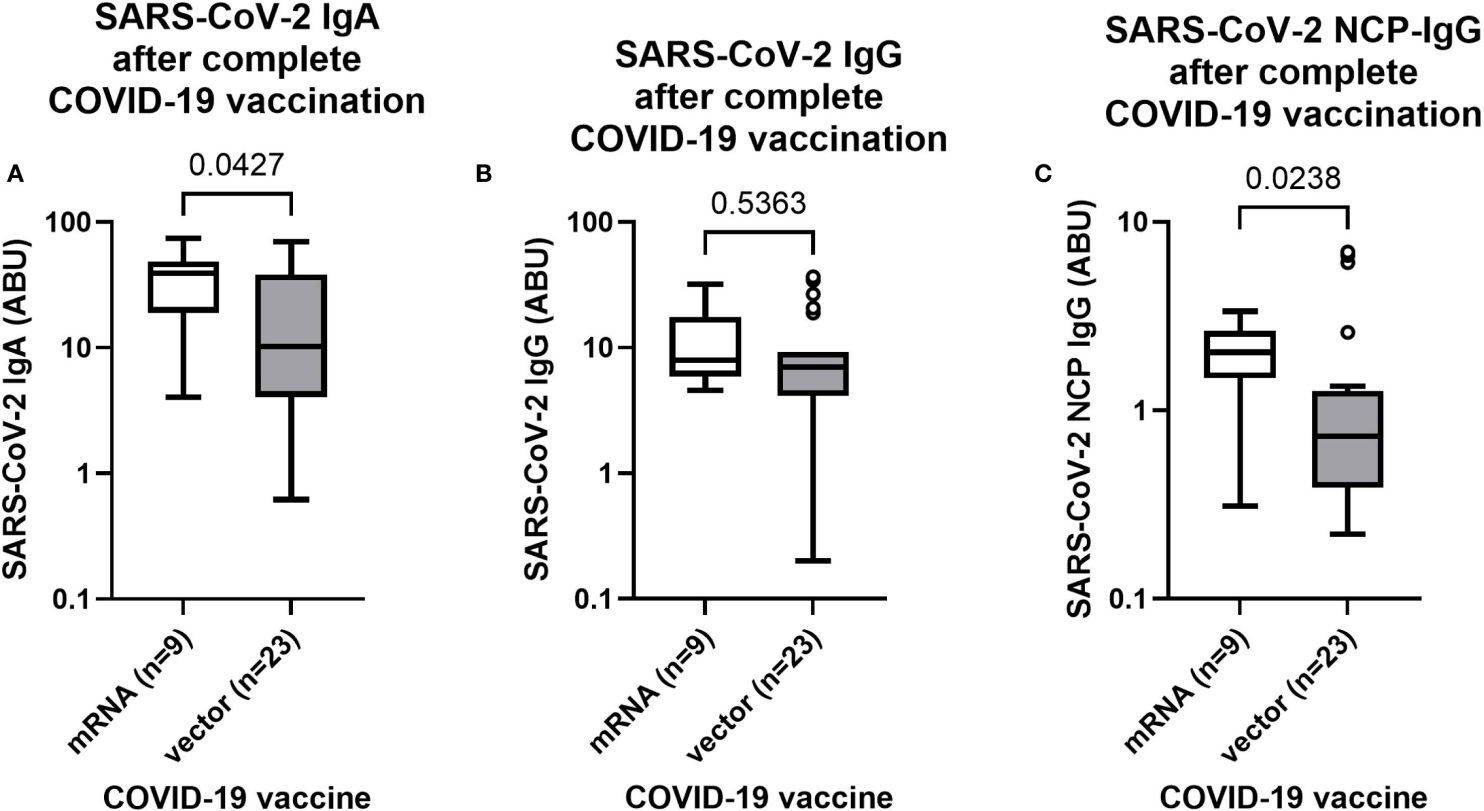
SARS-CoV-2 antibody response after complete vaccination with mRNA or vector-based COVID-19 vaccines in Ghanaian onchocerciasis infected individuals. SARS-CoV-2 spike IgA **(A)**, IgG **(B)** and nucleocapsid IgG **(C)** levels after complete vaccination (2 doses) with mRNA (white) (n=9) and vector-based (grey) (n=23) COVID-19 vaccines in Ghanaian onchocerciasis infected individuals who have not experienced any COVID-19 related symptoms during the pandemic. Indicated p-values were calculated using Mann-Whitney-U test. Bars represent the median ± IQR of antibody binding units (ABU). Significance is accepted if p <0.05.

After observation of elevated SARS-CoV-2 antibody levels in completely COVID-19 vaccinated individuals (**Figure 2**), differences between mRNA and vector-based COVID-19 were further investigated. The majority of complete (2 doses) COVID-19 vaccinated individuals received a vector-based COVID-19 vaccine (AstraZeneca or Johnson & Johnson), however more than 20% of complete vaccinated individuals received a mRNA vaccine, allowing a comparison of the antibody response following vaccination with different COVID-19 vaccines. Since clinical presentation of a COVID-19 infection is often associated with an elevated antibody response (43), only complete vaccinated individuals were taken into account who did not exhibit any COVID-19 related symptoms during the pandemic (n=32). Interestingly, mRNA vaccinated individuals (n=9) had notably higher (p=0.0427) SARS-CoV-2 IgA levels compared to the vector group (n=23) (**Figure 3A**). In contrast, SARS-CoV-2 IgG levels were statistically comparable (p=0.5363) among mRNA and vector vaccinated individuals (**Figure 3B**). However, remarkably elevated SARS-CoV-2 nucleocapsid IgG levels were detected (p=0.0238) in the mRNA vaccinated group, possibly indicating increased viral exposure compared to the vector vaccinated group (**Figure 3C**).

### 3.3 Diminished SARS-CoV-2 antibody response following COVID-19 vaccination in actively infected Ghanaian onchocerciasis-infected individuals

**Figure 4:**
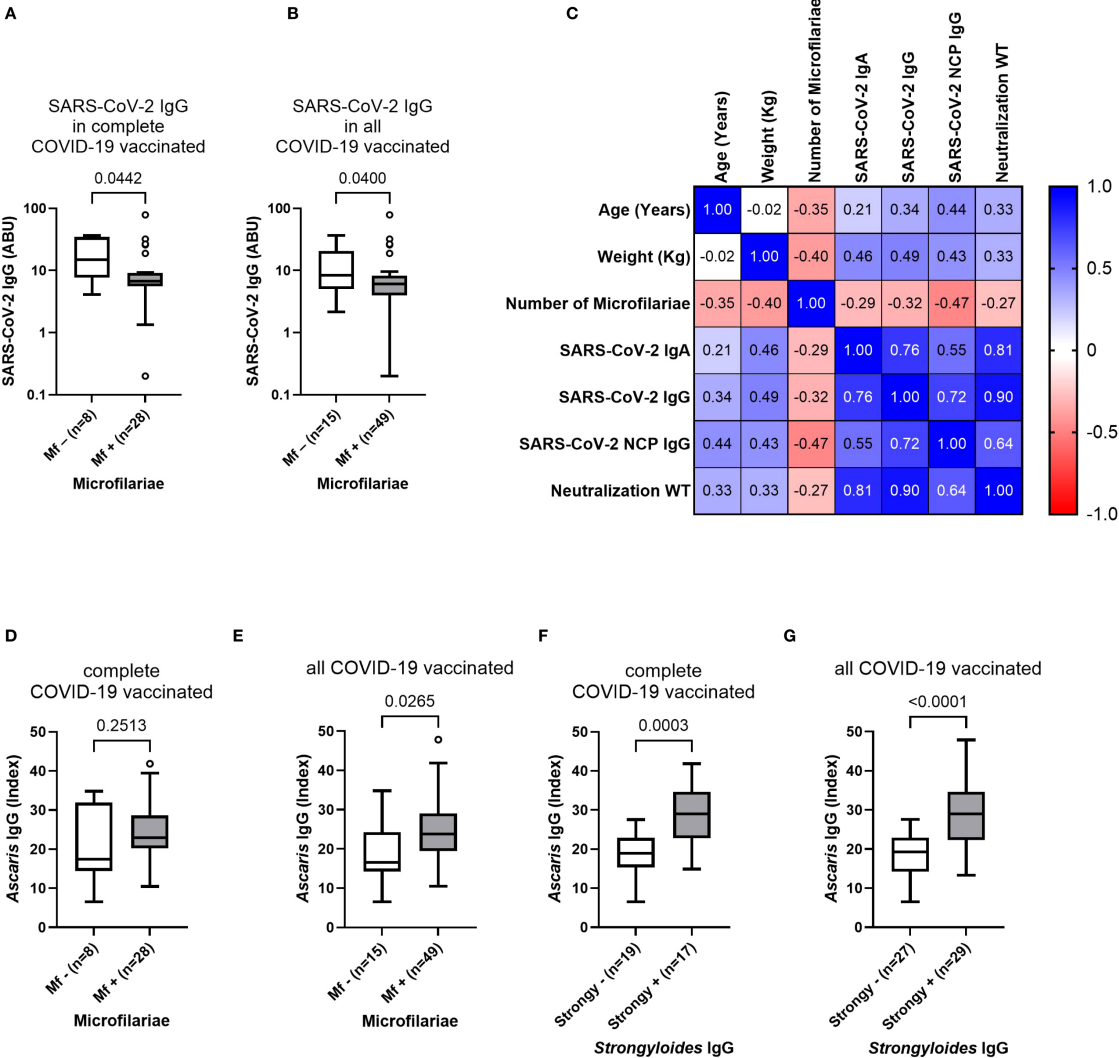
Influence of microfilaria positivity and load onto COVID-19 vaccine-induced antibody responses and soil-transmitted helminth co-infection seroprevalence. **(A, B)** show the SARS-CoV-2 specific antibody response in microfilaria negative (Mf–) and positive (Mf+) COVID-19 vaccinated Ghanaian onchocerciasis-infected participants. **(C)** shows correlations between microfilaremia and COVID-19 vaccine-induced antibody responses in incompletely vaccinated participants (n=22). **(D, E)** show *Ascaris lumbricoides* specific antibody titers in Mf –/+ complete **(D)** (n=8/n=28) and all **(E)** (n=15/n=49) COVID-19 vaccinated individuals. **(F, G)** show elevated *Ascaris*-specific IgG levels within *Strongyloides* seropositive (Strongy+) completely COVID-19 vaccinated **(F)** (n=17) and all COVID-19 vaccinated **(G)** (n=29) participants compared to the *Strongyloides* seronegative (Strongy–) group. Indicated p-values were calculated using Mann-Whitney U test. Bars represent the median ± IQR of antibody binding units **(A, B)** and Index values **(D–G)**. Correlations were performed using Spearman’s rank-order analysis **(C)**. Significance is accepted if p<0.05.

After observation of a robust antibody response following COVID-19 vaccination despite an underlying onchocerciasis infection, and increased antibody levels in COVID-19 vaccinated individuals compared to unvaccinated participants, the potential influence of the parasitic infection status and the corresponding microfilaremia onto the vaccine-induced antibody response was further investigated. For this purpose, the antibody response in completely (2 doses) and all COVID-19 vaccinated participants was compared based on their microfilaria status (negative/positive). Interestingly, the microfilaria positive completely COVID-19 vaccinated participants showed reduced (p=0.0442) SARS-CoV-2 IgG levels, compared to the microfilaria negative group (**Figure 4A**). A similar observation occurred in all COVID-19 vaccinated (incomplete + complete + booster) participants, showing reduced IgG levels within the Mf positive group (**Figure 4B**). In contrast, microfilaria positivity did not affect the IgA-mediated response, and comparable antibody levels were detected among microfilaria negative (Mf–) and positive (Mf+) COVID-19 infected and incompletely vaccinated individuals (**Supplementary Figure 2**). Nevertheless, by further investigating the potential effect of the microfilaremia onto the vaccine-induced responses, moderate negative correlations were found in incompletely COVID-19 vaccinated study participants (**Figure 4C**). The number of microfilariae negatively correlated with the SARS-CoV-2 spike-specific IgA (ρ= –0.29), spike-specific IgG (ρ= –0.32), nucleocapsid-specific IgG response (ρ= –0.47) and the SARS-CoV-2 neutralizing potential towards the wildtype (ρ= –0.27) (**Figure 4C**). In contrast, negative correlations of microfilaremia and vaccine-induced responses were not found in completely vaccinated study participants (**Supplementary Figure 3**). However, an important remark is the strong association between the microfilaria presence and soil-transmitted helminths (STH) seroprevalence (**Figures 4D-G**). Microfilaria positive individuals showed increased *Ascaris*-specific antibodies compared to the Mf negative group in both, completely and all COVID-19 vaccinated individuals (**Figures 4D, E**). Furthermore, the *Strongyloides* seropositive individuals showed also elevated *Ascaris*-specific antibodies (**Figures 4F, G**). Nevertheless, a multiplex PCR of stool samples revealed negative results for active gastrointestinal co-infections. Altogether it was observed that microfilaria positivity strongly correlated with STH-co-infection (*Ascaris* and *Strongyloides*) seroprevalence and further highlights the complex interplay between multiple helminths infection in endemic areas.

### 3.4 Reduced SARS-CoV-2 specific IgG1 and elevated SARS-CoV-2 IgG4 expression in helminth positive Ghanaian onchocerciasis-infected individuals

**Figure 5:**
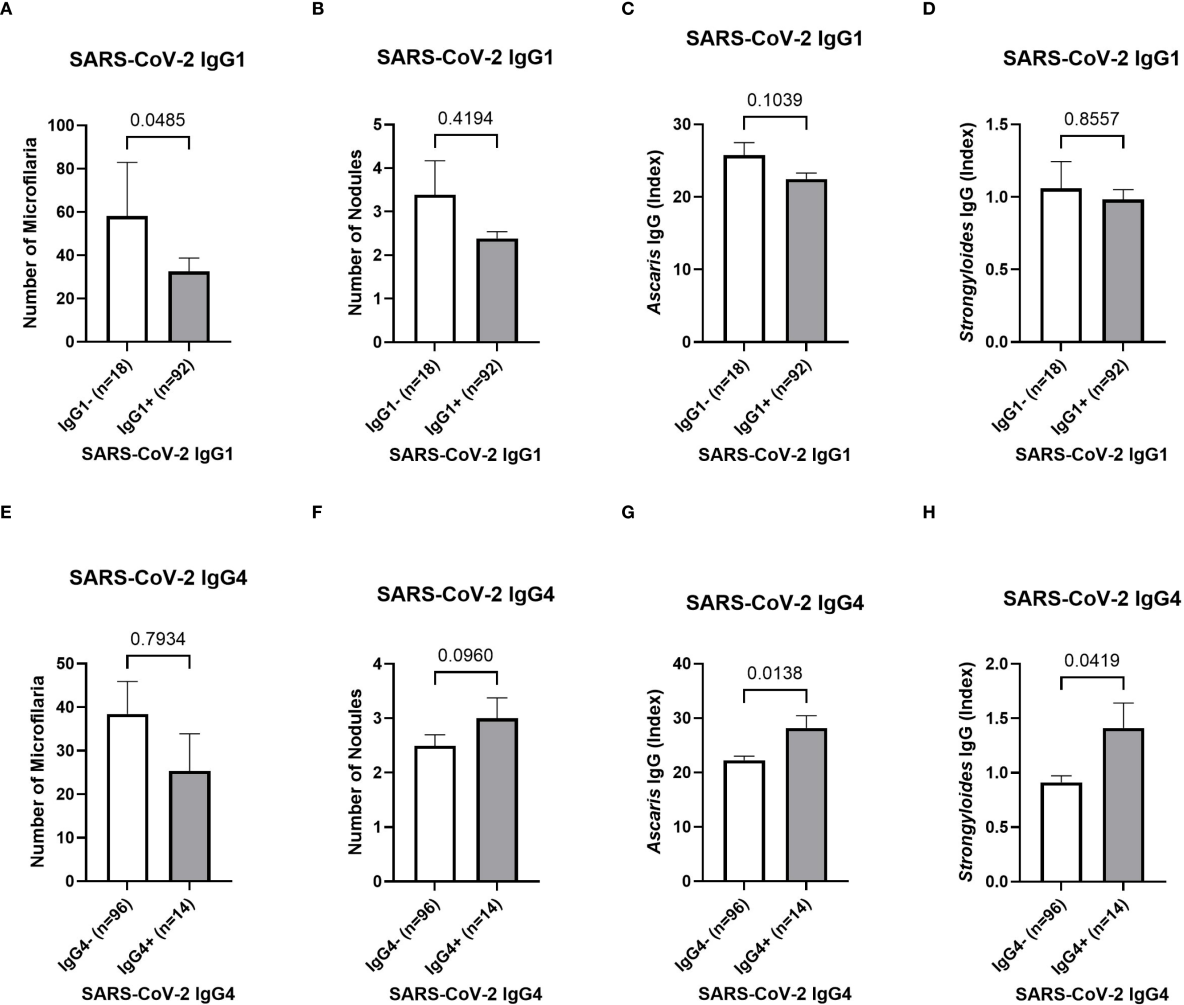
SARS-CoV-2 IgG subclass response depending on the parasitic status of Ghanaian onchocerciasis infected individuals. The potential influence of the microfilaremia **(A, E)**, number of onchocercomas **(B, F)**, *Ascaris* seropositivity **(C, G)** and *Strongyloides* seropositivity **(D, H)** onto SARS-CoV-2 specific IgG1 **(A-D)** and IgG4 **(E-H)**. Significantly (p=0.0485) higher number of microfilariae within the SARS-CoV-2 IgG1 positive (grey) (n=18) individuals compared to the negative (white) (n=92) group **(A)**. Statistically significant elevated *Ascaris* and *Strongyloides* antibody titers within SARS-CoV-2 IgG4 positive (grey) (n=14) group compared to the negative (white) (n=96) group **(G, H)**. Indicated p-values were calculated using Mann-Whitney U test. Bars represent the mean ± SEM of the number of microfilaria **(A, E)**, number of nodules **(B, F)**, *Ascaris*-specific antibody index values **(C, G)** and *Strongyloides*-specific index values **(D, H)**. Significance is accepted if p<0.05.

After analyzing the effect of the parasitic status on the COVID-19 vaccine-induced antibody responses, a potential impact onto the systemic cytokine and chemokine profile was further analyzed via a multiplex immunoassay. However, no effect has been observed and comparable levels of Th1, Th2, and regulatory T cell-related cytokines were detected within the plasma of Ghanaian onchocerciasis infected individuals irrespective of their filarial status (**Supplementary Figure 4**). In the next step, the potential impact of helminth seropositivity onto the SARS-CoV-2 specific IgG subclass response was further investigated. Since the majority (58.2%) of all onchocerciasis-infected participants (n=110) received at least one COVID-19 vaccine dose, and the majority of unvaccinated individuals showed SARS-CoV-2 antibody seropositivity (86% IgA, 77% IgG), indicating exposure to the circulating agent, the SARS-CoV-2 IgG subclasses: IgG1, IgG3 and IgG4 were further investigated in all participants by using a designed ELISA. The influence of the following factors onto the IgG subclass response was evaluated: number of microfilariae, number of onchocercomas (nodules), *Ascaris lumbricoides* seropositivity and *Strongyloides stercoralis* seropositivity. The number of microfilariae was significantly reduced (p=0.0485) in those participants, exhibiting a SARS-CoV-2 IgG1 response (IgG1+) compared to the negative (IgG1–) group (**Figure 5A**). The number of nodules and STH-specific antibodies did not differ between SARS-CoV-2 IgG1 negative and positive participants (**Figures 5B-D**). No influence of previous mentioned parasitic factors onto SARS-CoV-2 IgG3 response was observed (**Supplementary Figure 5**). Interestingly, significantly elevated *Ascaris* and *Strongyloides* antibody levels (p=0.0138/p=0.0419) were observed within SARS-CoV-2 IgG4 positive individuals compared to the negative group (**Figures 5G, H**). Finally, since all participants were part of a clinical trial, the potential effect of deworming treatment onto the COVID-19 vaccine efficacy and the corresponding SARS-CoV-2 antibody response was analyzed. However, the treatment revealed no remarkable differences within the SARS-CoV-2 specific antibody response among COVID-19 vaccinated onchocerciasis-infected participants (**Supplementary Figure 6**).

## 4 Discussion

The early development and global distribution of various COVID-19 vaccines had a significant impact on the pandemic by reducing disease severity and associated mortality (44). Helminth infections are among the most common infections worldwide, affecting almost one quarter of the world’s population (42). Helminths are known for their ability to modify vaccine-induced responses of their host (30). Due to the high prevalence of helminths, investigating the potential effect on COVID-19 vaccine efficacy, plays a major role in future vaccine development and vaccination campaigns in regions such as sub-Saharan Africa. Therefore, the present study explored the humoral immune response following vaccination with different COVID-19 vaccines in onchocerciasis infected Ghanaian individuals. The seroprevalence analyses revealed that the majority of COVID-19 unvaccinated participants was tested SARS-CoV-2 antibody seropositive (86% for IgA, 77% for IgG), indicating viral exposure to the causative agent at the sampling time point (December 2022). These high seroprevalence rates are consistent with other prevalence studies among African countries (45–49). In this study, the SARS-CoV-2 spike (S) and nucleocapsid (NCP) protein seroprevalence allowed differentiation between vaccine- and infection-induced immunity since all study participants received COVID-19 vaccines, containing only the genetic information for the spike protein. A lower but still remarkable portion of nucleocapsid seropositivity was detected among incompletely (1 dose) and completely (2 doses) COVID-19 vaccinated participants (63% and 50%, respectively), compared to unvaccinated study subjects, indicating a prior or breakthrough infection. These findings indicate a high degree of SARS-CoV-2 exposure among both COVID-19 unvaccinated and vaccinated individuals, which contributes to the understanding of the viral spread in rural areas within the western-north region of Ghana. Furthermore, a strong vaccine-induced antibody response following COVID-19 vaccination was shown in Ghanaian onchocerciasis infected individuals. The levels of SARS-CoV-2 specific antibodies (IgA and IgG) were highest in completely vaccinated (2 doses) individuals, highlighting the robustness of the vaccine despite an underlying filarial infection. The incompletely vaccinated (1 dose) participants also showed an elevated antibody response compared to the unvaccinated and seronegative control group. Interestingly, the healthy endemic controls showed a significantly reduced SARS-CoV-2 IgA response compared to the completely vaccinated onchocerciasis-infected individuals as well as statistical comparable SARS-CoV-2 IgG levels. Moreover, all groups show a notable variability within the antibody titers. This could be explained by the broad time frame of the COVID-19 vaccination date (between January 2021 and December 2022) and the variability within the different COVID-19 vaccines that were administered which complicate the comparison and evaluation of vaccine-induced responses. Nevertheless, it could be stated, that mRNA-based (BioNTech-Pfizer and Moderna) and vector-based (AstraZeneca and Johnson & Johnson) COVID-19 vaccines induced a potent antibody response in onchocerciasis infected individuals, and statistically equivalent antibody levels compared to the healthy controls.

Another interesting aspect of this work is the neutralizing potential of SARS-CoV-2-specifc antibodies towards the wildtype and five variants of concern including Alpha (B.1.1.7), Beta (B.1.351), Gamma (P.1), Delta (B.1.617.2) and Omicron (B.1.1.529). Only completely vaccinated participants showed significantly elevated neutralizing antibodies towards the wildtype, compared to the other groups (unvaccinated and incomplete) and the other variants. A possible explanation for that observation might be waning immunity and increased immune evasion of variants (50).

A notable variation within the vaccine-induced antibody response following vaccination with different COVID-19 vaccines has been shown previously (51). For that reason, the antibody response of mRNA-based and adenovector-based vaccines was compared after complete (2 doses) vaccination. Both vaccines induced comparable SARS-CoV-2 specific IgG levels in onchocerciasis infected individuals Interestingly, the mRNA vaccinated group showed elevated IgA levels compared to the vector group. The higher IgA levels could either derive from a stronger response of the mRNA vaccine, or a natural infection since IgA is the most abundant isotype on mucosal surfaces and often induced by viral exposure (52). This hypothesis is supported by the fact, that the mRNA group significantly increased NCP-specific antibody titers had which could only derive from a natural infection. This finding could imply that mRNA vaccinees may be more susceptible to a SARS-CoV-2 infection in contrast to those who received a vector-based vaccine. This would be contrary to other studies, which showed that mRNA vaccines usually induce higher antibody responses compared to the vector-based vaccines (51). A plausible explanation might be the nature of the vaccines itself. The ChAdOx1 (AstraZeneca) and Ad26.CoV2.S (Johnson & Johnson) vaccines use an adenovirus vector that encodes the SARS-CoV-2 spike protein (21). In contrast, BioNTech’s (BNT162b2) and Moderna’s vaccine (mRNA-1273) rely on spike-protein encoding mRNA, which encapsulated in lipid nanoparticles (53). Adenovirus vectored and mRNA vaccines have shown to induce a strong type 1 (Th1) cellular response, with levels of IFNγ-secreting T cells (21). The adenovirus vectored vaccines mimicry the natural course of the infection to a certain extend (21), which might induce a more robust humoral response in filarial positive individuals compared to the mRNA-based vaccines. It might be that adenovirus vectored vaccines are less affected by the helminth-induced immunomodulation and better overcome a type 2 response which is usually present in helminth-positive individuals. Nevertheless, further studies with larger cohorts and a higher sample size of mRNA vaccinated individuals are required to confirm the findings obtained in the present work.

The parasitic status and helminths co-infection history, and its possible impact on COVID-19 vaccine efficacy, was investigated by several tests including: nodulectomy, skin biopsies, serological assays and rapid tests. It has to be stated that all participants were part of a clinical trial and received treatment 18 months prior to sample collection, and despite the fact that a nodulectomy was performed, the assessment and evaluation of the viability and number of adult worms present in those nodules was not possible within the present study. However, the majority (74%) of participants were tested positive for microfilaria, indicating an active infection with reproducing adult worms. Interestingly, the completely vaccinated participants which were microfilaria positive, showed reduced SARS-CoV-2 IgG levels compared to the microfilaria negative group. In contrast, other groups (COVID-19 infected, incompletely vaccinated) and SARS-CoV-2 IgA levels were not affected by the presence of microfilaria. This finding may indicate that an active onchocerciasis infection and the filarial load could negatively correlate with the vaccine-induced antibody response following COVID-19 vaccination.

However, it has to be considered that the presence of microfilariae was strongly associated with seropositivity for soil-transmitted helminths (STH) including *Ascaris* and *Strongyloides*. Participants which were microfilaria positive, showed significantly higher STH-specific antibody titers compared to the microfilaria negative group. This observation might either be explained by filaria cross-reactivity of the serological assays, or that an active onchocerciasis infection and a high microfilaria load contributes to a higher susceptibility for co-infections. Since all participants were tested negative for active gastrointestinal helminth co-infections, prior co-infections may contribute to a reduced COVID-19 vaccine-induced response in filarial positive individuals. The complex interaction of chronic filarial infections, co-infections and polyparasitism further complicates the analysis. This finding is important and might indicate that deworming campaigns may improve COVID-19 vaccine responses of co-infected populations in helminth-endemic regions. A possible explanation for a reduced COVID-19 vaccine-induced response, might be the strong type 2 (Th2) immune response which is induced by helminths and characterized by the production of cytokines such as IL-4, IL-5, and IL-10 (41). Helminths are known for their potential to modulate vaccine responses (41). It might be, depending on the parasitic status and the filarial load, that the strong helminth-induced Th2 response overrides the vaccine-induced Th1 response, resulting in a diminished immunization. The systemic cytokine and chemokine profile was investigated, to further explore the influence of helminths and their possible immunomodulatory effect. Nevertheless, statistically comparable levels of Th1, Th2 and regulatory T cell-related cytokines were detected, regardless of the parasitic status. Since all participants were part of a clinical trial, the possible effect of treatment onto the vaccine efficacy was further investigated. The participants were grouped into the following four treatment arms: (1) placebo, (2) albendazole, (3) rifampicin and albendazole (for 7 days) and (4) rifampicin and albendazole (for 14 days). However, statistical comparable COVID-19 vaccine-induced antibody levels were observed among all treatment groups. Based on these findings, we do not see evidence supporting the need for pre-treatment for helminths to enhance immunogenicity. Nonetheless, we acknowledge that further studies with larger cohorts and mechanistic insights would be valuable to confirm these findings across different settings.

In the last step of this work, the possible effect of helminths onto the SARS-CoV-2 IgG subclass response following vaccination and/or natural infection, was further explored. Since almost all participants were SARS-CoV-2 antibody seropositive due to vaccination or infection, they were not further grouped according to their vaccination status but instead according to their positivity and detectable levels of certain IgG subclasses in relation to their parasitic status. Several studies have shown that certain IgG subclasses are related to excessive inflammation and COVID-19 severity (54–59). IgG1 and IgG3 are the subclasses with the highest potential to activate the immune system, while IgG4 has less activating potential and can instead inhibit effector functions (60). It has been shown that SARS-CoV-2 specific IgG1 and IgG3 were associated with COVID-19 disease pathology (56, 58, 59). In contrast, IgG4 is considered as an anti-inflammatory immunoglobulin, and there are contradictory suggestions whether increased IgG4 levels are beneficial or detrimental regarding the disease outcome (54). In the present study, those participants which did not express SARS-CoV-2 IgG1 showed a higher number of microfilariae compared to the IgG1 positive group. The number of nodules or the seropositivity for soil-transmitted helminths (STH) did not show any influence on the SARS-CoV-2 IgG1 subclass response. None of the parasitic tests showed any influence on the vaccine- or infection-induced SARS-CoV-2 IgG3 response. Interestingly, those participants who exhibited a SARS-CoV-2 specific IgG4 response showed significantly higher STH-specific antibodies compared to the IgG4 negative group. The elevated IgG4 response is likely linked to a type 2-skewed immune profile in helminth-positive individuals. It has been shown previously, that the helminth-induced regulatory response is associated with induction of IgG4 (61–63). Moreover, it was shown that Th2 cytokines are able to stimulate B cells for IgG4 production (64). A lack of SARS-CoV-2 IgG1 and high expression of IgG4 in helminth positive individuals may contribute to reduced disease severity and mortality in endemic regions. The potential immunomodulatory influence of helminths onto the SARS-CoV-2 IgG subclass response may present another contributing factor on the unexpectedly mild COVID-19 pandemic in sub-Saharan Africa.

There is currently no data available, whether filarial infection influences COVID-19 vaccine efficacy, which further highlights the relevance of the present work and the need for further investigation. However, a few human studies have previously investigated the impact of onchocerciasis on immune responses following tetanus vaccination (32, 33, 35). One study showed diminished cell-mediated and humoral responses following tetanus immunization in onchocerciasis-infected individuals (35). This was partially confirmed by another study which showed an impaired cellular response following tetanus vaccination in patients with generalized onchocerciasis, although humoral responses remained unaltered (33). Interestingly, a third study showed that *O. volvulus* infection does not prevent the development of a protective antibody response following tetanus vaccination, however, those with heavier infections showed an impaired response compared to the light infection group (32). These finding aligns to a certain extent with that of the present study, showing that the microfilaria presence and load as well as prior STH-co-infections may lead to reduced COVID-19 vaccine-induced antibody response. Studying vaccine responses in helminth infected populations is highly important for future vaccine development since it helps better understanding immune modulation in real-world settings. Helminth-induced immunomodulation mimics to a certain extent other immune-modulating conditions such as malnutrition, HIV, aging and could present thereby a valuable model for other conditions as well. Furthermore it could help to improve the vaccine design for immunosuppressed or modulated populations in terms of: adjuvants, delivery platforms or dosing strategies, especiallyby taking into account, that future vaccines against other infectious diseases might also rely on strategies that have been used for the COVID-19 vaccines, such as mRNA or vector-based vaccines. In addition, it could help to improve co-intervention strategies such as deworming campaigns prior to vaccination.

One limitation of the present work is the broad time frame within the vaccination date of the participants, which complicates the evaluation of vaccine responses due to waning immunity over time. Another key limitation is the absence of confirmed data on prior SARS-CoV-2 infections before enrollment. Although previous exposure through serological marker – specifically, the presence of spike- and nucleocapsid-specific antibodies – has been investigated, and detailed self-reported information on COVID-19 related symptoms was collected, we cannot exclude the possibility of non- responder or asymptomatic infections. This could underestimate the true spread of the virus and complicates the evaluation of vaccine efficacy in terms of incidence or clinical presentation. However, the documentation of COVID-19 related symptoms revealed that only a minority of study subjects (11%) exhibited any flu-like symptoms and none participant suffered of severe COVID-19 related to hospital admission. Another limitation was the small sample size of healthy endemic controls, and discrepancies in the vaccination dates, nevertheless, their inclusion enabled a meaningful reference for evaluating potential parasite-associated modulation of vaccine-induced antibody responses. Despite the fact that all healthy endemic controls were tested negative for onchocerciasis, the presence of undetected co-infections and the undeterminable infection history further complicated the analysis, especially by using serological tests for the detection of helminths infections. This further highlights the complications within the evaluation of vaccine efficacy and the complex interplay of contributing factors such as pre-existing infections and co-infections. Furthermore, the present study contained a gender bias, which is skewing towards men. There was no gender effect observed in the present data, which aligns with previously reported data, where age was shown as dominating factor in vaccine-induced responses instead of the gender (51). Regarding the tests that were involved in the analyses, it has to be mentioned that a cell-and virus-free ACE-2 competition assay was used as neutralization assay, allowing a multiplex readout performable under biosafety level 2 conditions. Another important note is that we were not able to detect SARS-CoV-2-specific IgG2 levels within our ELISA. Serological assays for the detection of helminth infection have to potential to detect current and past infections, extending the validity and robustness of the present work. Finally, while the present study focused on the humoral immune response to COVID-19 vaccines, cellular responses also play a key role (65) and further investigations of the potential effect of helminths on the COVID-19 vaccine-induced cellular responses are required to fill this knowledge gap and delve deeper into the involved helminth-induced immunomodulatory mechanisms.

## 5 Conclusion

In summary, the present study concludes that the COVID-19 vaccines induce a robust antibody response and neutralizing antibodies towards the wildtype after complete vaccination despite onchocerciasis as underlying helminth infection. However, a lack of variant-specific neutralizing antibodies, due to waning immunity and immune evasion of the variants, may led to an increased susceptibility to a SARS-CoV-2 infection. The post-vaccination SARS-CoV-2 IgG antibody titers induced by vector- and mRNA-based vaccines were comparable, but elevated IgA and NCP-IgG antibodies could indicate a higher breakthrough infection rate in mRNA vaccinated individuals. Participants with an active onchocerciasis infection (microfilaria positive) showed a reduced vaccine-induced antibody response following complete COVID-19 vaccination, which may imply that the parasitic status and filaria load could lead to a diminished vaccine response. Nevertheless, microfilaria positivity was strongly associated with soil-transmitted helminth (STH) seropositivity, which could also contribute to a hampered vaccine response. The parasitic status may contribute to a modified SARS-CoV-2 IgG subclass response, since microfilaria and STH-seropositivity were associated with a lack of pro-inflammatory IgG1 and increased anti-inflammatory IgG4 levels. This finding might contribute to the high number of asymptomatic or mild cases and the relatively mild course of the COVID-19 pandemic in sub-Saharan Africa.

## Data Availability

The raw data supporting the conclusions of this article will be made available by the authors, without undue reservation.
