# Supplementary material for: Humoral immune response to COVID-19 vaccines in onchocerciasis-infected individuals: a field study from Ghana
Source: Front Immunol. 2025 Oct 1;16:1633187. doi: 10.3389/fimmu.2025.1633187 (PMC12521167; doi:10.3389/fimmu.2025.1633187)
Supplement: Supplementary file 1 [file Table1.docx]

Supplementary Material

Table 1. COVID-19 vaccination time frame of Ghanaian onchocerciasis study participants

| Month | 01 | 07 | 10 | 11 | 12 | | 01 | 02 | 03 | 04 | 06 | 07 | 09 | 10 | 11 | 12 | |
| --- | --- | --- | --- | --- | --- | --- | --- | --- | --- | --- | --- | --- | --- | --- | --- | --- | --- |
| Year | 2021 | | | | | 2022 | | | | | | | | | | |  |
| 1^st^ Dose (n=) | 1 | 1 | 8 | 13 | 9 | | 3 | 22 | - | - | 4 | - | - | 1 | - | - | |
| 2^nd^ Dose (n=) | - | - | - | 5 | 7 | | 1 | 7 | 4 | 8 | 5 | 2 | - | 1 | 2 | - | |
| 3^rd^ Dose (n=) | - | - | - | - | - | | 1 | - | - | - | 2 | - | 1 | 1 | - | 1 | |

Table 2. Demographic and clinical characteristics of healthy Ghanaian endemic controls (HEC)

| Sample Size (n=) | 12 |
| --- | --- |
| Age (Min-Max, Mean ± SD) | 23 – 58 (34.9 ± 10.1) |
| Sex (M / F) | 11 (92%) / 1 (8%) |
| Hypertension (n=) | 1 (7%) |
| BMI (Mean ± SD) | 24.7 ± 2.7 |
| COVID-19 vaccination (Yes / No) | 10 (83%) / 2 (17%) |
| AstraZeneca / Johnson & Johnson | 4 (40%) / 6 (60%) |
| Confirmed SARS CoV-2 infection | 1 (8%) |
| Adverse Events 1^st^ vaccination  (No / Mild / Severe) | 2 / 7 / 1  20% / 70% / 10% |
| Adverse Events 2^nd^ vaccination  (No / Mild / Severe) | 3 / 1 / 0  75% / 25% / 0% |

#
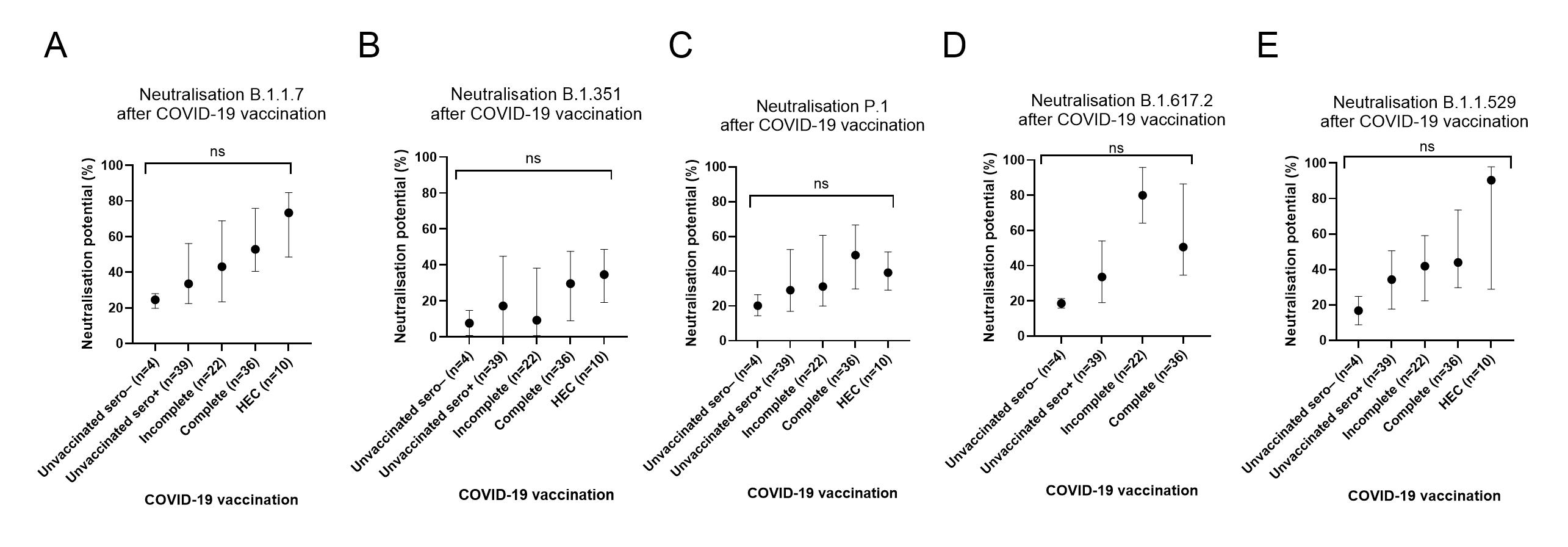
Comparable levels of SARS-CoV-2 neutralizing antibodies towards the variants of concern in COVID-19 vaccinated Ghanaian onchocerciasis infected individuals

Figure 1. Neutralizing potential of SARS-CoV-2 specific antibodies within COVID-19 vaccinated Ghanaian onchocerciasis infected participants. Neutralizing antibody levels towards five variants of concern including Alpha (B.1.1.7) (A), Beta (B.1.351) (B), Gamma (P.1) (C), Delta (B.1.617.2) (D) and Omicron (B.1.1.529) (E) were determined. Neutralizing antibody responses were compared among onchocerciasis participant groups based on their COVID-19 vaccination status: unvaccinated and seronegative (n=4), unvaccinated and seropositive (n=39), incompletely (1 dose) vaccinated (n=22), completely (2 doses) vaccinated (n=36), and vaccinated healthy endemic controls (HEC) (n=10). Indicated p-values were calculated using Kruskal-Wallis’ test followed by Dunn’s post hoc test for group comparison. Dots represent the median ± IQR of antibody neutralizing potential (%). Significance is accepted if p <0.05.

#
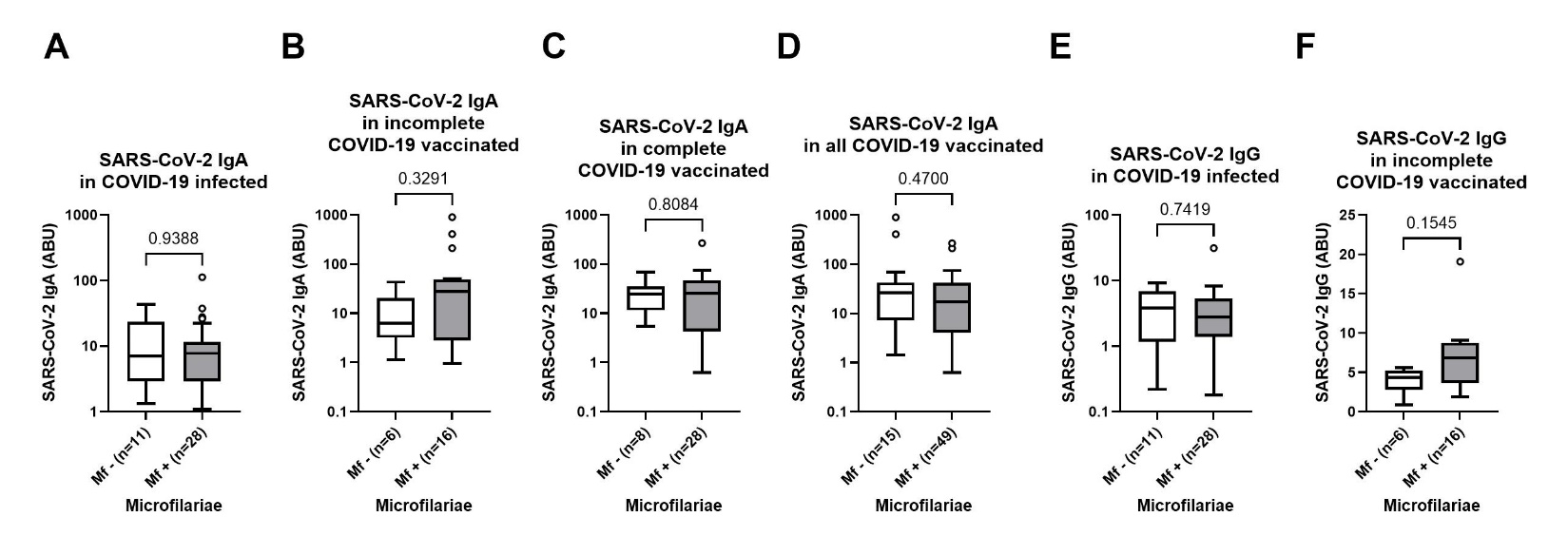
Comparable antibody responses following COVID-19 vaccination and/or natural infection irrespective of the microfilaremia of Ghanaian onchocerciasis infected individuals

Figure 2. SARS-CoV-2 specific antibody response in microfilaria negative and positive COVID-19 infected and vaccinated Ghanaian onchocerciasis participants. Comparable SARS-CoV-2 specific IgA (A-D) and IgG (E+F) levels within microfilariae (Mf) negative (white) and positive (grey) COVID-19 infected (A+E), incomplete (B+F), complete (C) and all (D) COVID-19 vaccinated participants. Indicated p-values were calculated using Mann-Whitney-U test. Bars represent the median ± IQR of antibody binding units (ABU). Significance is accepted if p <0.05.

# No influence of the parasitic status on the systemic cytokine and chemokine profile of Ghanaian onchocerciasis infected individuals

Figure 3. No effect of parasitic status on the systemic cytokine and chemokine profile of onchocerciasis individuals. Comparable levels of Th1 (A-D), Th2 (E-H), and Treg-related (I-L) cytokines were detected in Ghanaian onchocerciasis infected participants irrespective of their filarial status including: microfilaria load (A+E+I), number of nodules (B+F+J), *Schistosoma* seropositivity (C+G+K), or *Strongyloides* seropositivity (D+H+L). Indicated p-values were calculated using Mann-Whitney U test. Bars represent the mean ± SEM of cytokines and chemokines (pg/ml). Significance is accepted if p <0.05.


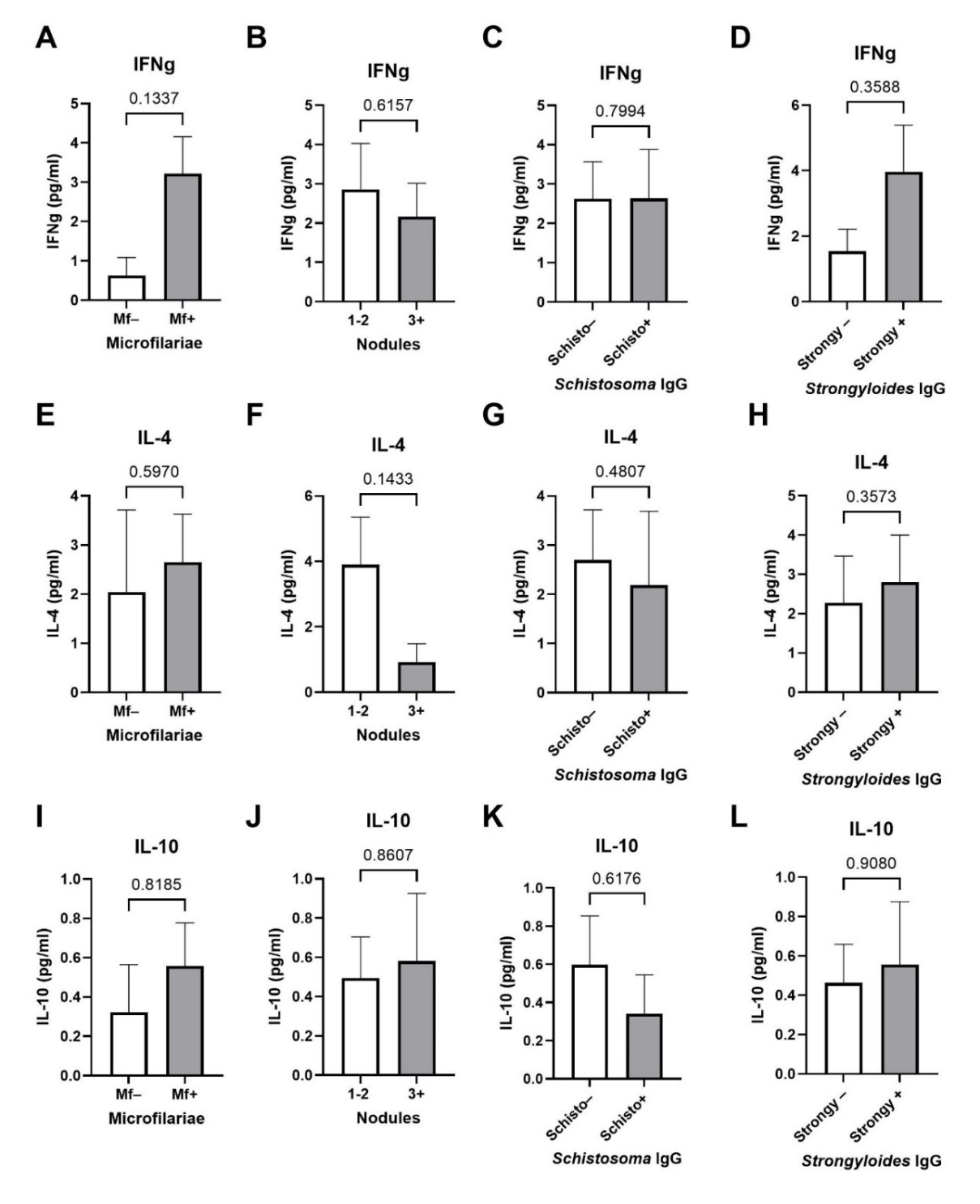


#
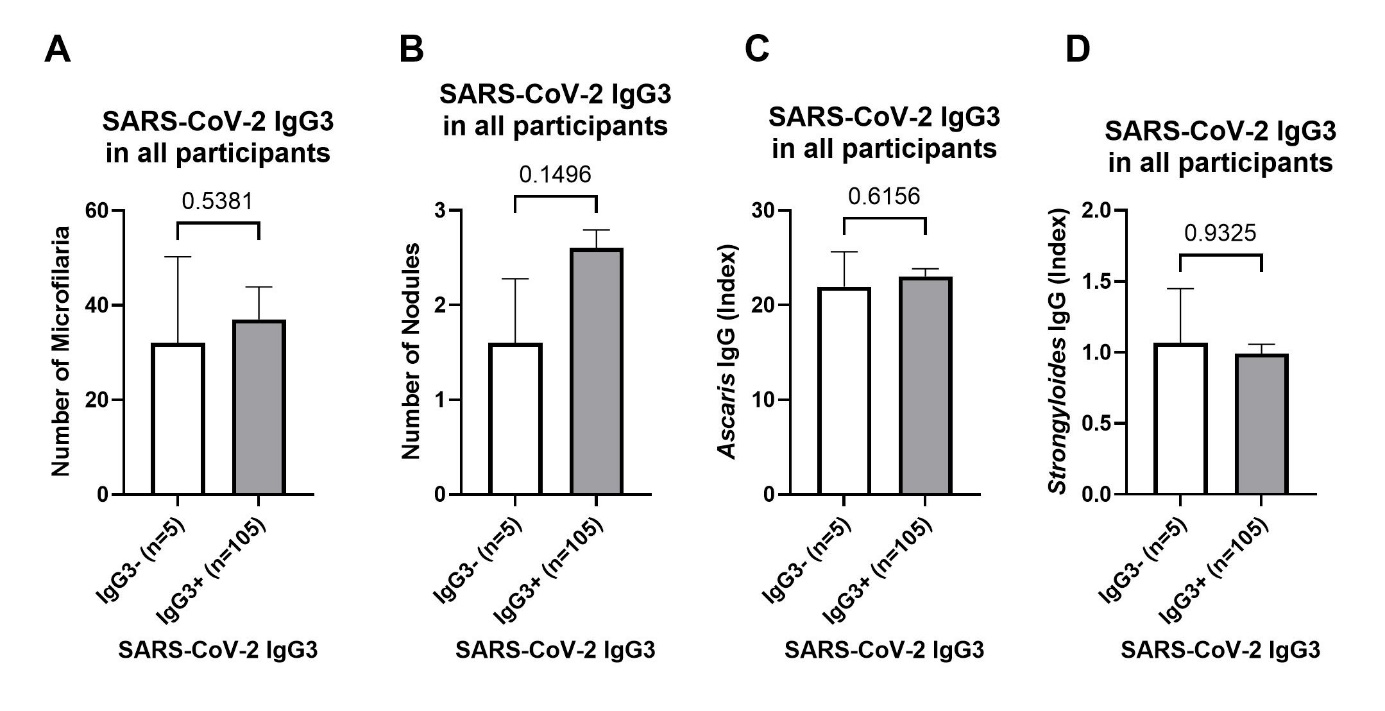
Comparable SARS-CoV-2 specific IgG3 subclass response irrespective of the parasitic status of Ghanaian onchocerciasis infected individuals

Figure 4. SARS-CoV-2 specific IgG3 subclass response depending on the parasitic status of Ghanaian onchocerciasis infected individuals. No influence of the microfilaremia (A), number of onchocercomas (B), *Ascaris* seropositivity (C) and *Strongyloides* seropositivity (D) onto the SARS-CoV-2 specific IgG3 subclass response. Indicated p-values were calculated using Mann-Whitney U test. Bars represent the mean ± SEM of the number of microfilaria (A), number of nodules (B), *Ascaris*-specific antibody index values (C) and *Strongyloides*-specific index values (D). Significance is accepted if p<0.05.

#
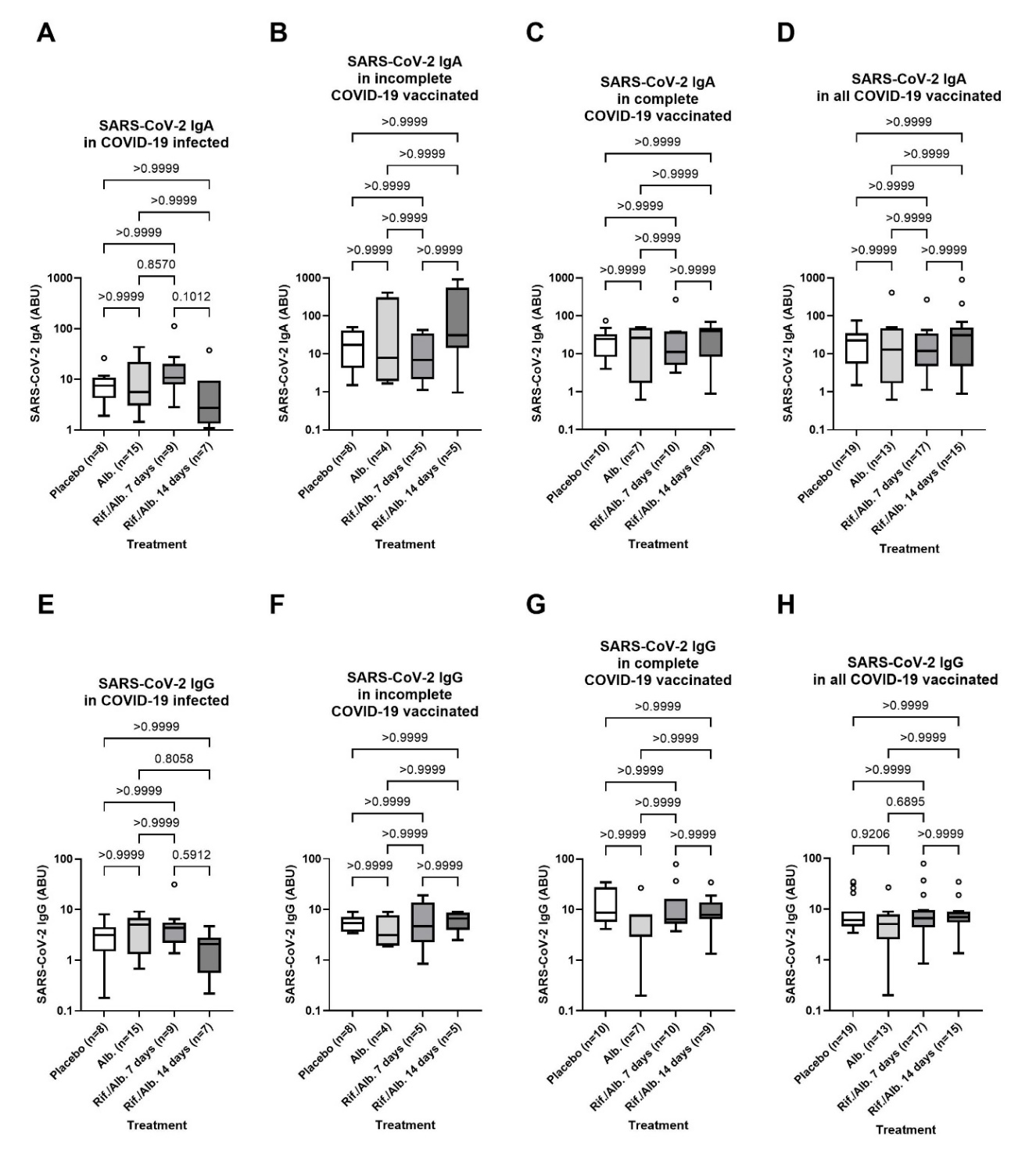
No effect of prior treatment onto SARS-CoV-2 specific antibody response in COVID-19 vaccinated onchocerciasis Ghanaian individuals

Figure 5. SARS-CoV-2 antibody response in COVID-19 vaccinated onchocerciasis participants with varying treatment administration 18-months prior to sample collection. Comparable SARS-CoV-2 Spike-specific IgA (A-D) and IgG (D-F) antibody levels among COVID-19 infected (n=39) (A+E), incompletely (n=22) (B+F), completely (n=36) (C+G) and all (n=64) COVID-19 vaccinated (D+H) individuals independent of treatment with albendazole (light blue), rifampicin + albendazole (7 days) (blue) and rifampicin + albendazole (14 days) (dark blue) 18-months prior to blood sampling compared to the placebo group (white). Indicated p-values were calculated using Kruskal-Wallis’ test followed by Dunn’s post hoc test for group comparison. Bars represent the median ± IQR of antibody binding units (ABU). Significance is accepted if p <0.05
